# Sterility and Its Treatment1Read before the Buffalo Academy of Medicine, March 22, 1904.

**Published:** 1904-05

**Authors:** Herman E. Hayd

**Affiliations:** Buffalo, N. Y.


### BUFFALO MEDICAL JOURNAL.

Vol. XLIII.—LIX. MAY, 1904. No. 10

### ORIGINAL COMMUNICATIONS.

### Sterility and Its Treatment.[1]

By HERMAN E. HAYD, M. D., M. R. A. S., Eng., Buffalo, N. Y.

THE introductory chapter in Coe and Keating's Gynecology is from the pen of the late William Goodell, and to those who have not read it I commend it, because I think it is one of the classics of modern medicine. He says, that undoubtedly some of the worst forms of women's diseases come from the three following causes: the specific infection of wives by their husbands; criminal abortion; and the prevention of conception. So long as society condones in a man such lapses of virtue as it peremptorily and pitilessly condemns in woman, so long as a chaste woman is willing to take to herself an unchaste husband, so long will young men indulge in illicit sexual pleasures. Sterility, miscarriages, oöphoritis, salpingitis of every kind and degree, pelvic and intestinal adhesions, chronic ill health, and even death must necessarily result from such marital unions.

The article in the *American Textbook of Gynecology,* from which I have taken much of my paper says, sterility in the female implies an inability to bring forth a living child. This condition may be either relative or absolute,—relative when there is simply diminished procreative power, and absolute when procreation is impossible. Sterility may be congenital, in which case it results from a faulty development of the organs necessary for procreation. It is more often acquired, and is then due to diseases involving one or more of the organs necessary for the process of genesis.

Matthews Duncan says that one marriage in every ten in Great Britain is sterile. Statements of this character are, as a rule, valueless, because many women are childless from intentional causes during early married life, and when the inevitable

1. Read before the Buffalo Academy of Medicine, March 22, 1904.

yearning for offspring comes, like Rachel, they are not comforted. Women do not ordinarily conceive during the nursing period, or after the climacteric; and this condition is spoken of as physiological sterility. Again, many women conceive, but are not able to carry the ovum through the necessary period of uterogestation from diseases of the uterus and its lining membrane, or causes inherent in the ovum.

For the process of generation in the female, various organs are necessary, and upon their degree of development and tonicity results fecundity or absolute or relative sterility. Many men are sterile; and some authorities estimate the percentage quite high, but this phase of the subject need not interest us especially this evening, as the fertility of the male fluid can be easily and quickly ascertained by examining the number, size and vitality of the contained spermatozoa, and if they are found to be normal fecundation should result from sexual union in a healthy woman.

An interesting experience occurred to me during the past few weeks, but, as it had happened before, I was on the alert. Two years ago a young bride consulted me on account of certain distressing symptoms about her pelvic organs. Upon examination, there was found an erosion of the cervix and cervical and uterine catarrh. She was curetted at the German Hospital, and, so far as her feelings were concerned, she was well and happy. After two years of married life without children, she again called, to find the cause of her sterility. Upon examination, I found her organs well and healthy, and I suggested that she send her husband to me, who is a large, fine, healthy, young man. He gave me a history of having had gonorrhea five years previous to marriage, and with the attack he had double orchitis. I had him bring me some of his seminal discharge, collected by means of an ordinary condom, and I found it filled with prostatic crystals, but absolutely no spermatozoa.

Many women have been subjected to needless pain and operations for the cure of sterility, when the cause existed in the husband and could be so fully established by such simple means. However, even when nature has provided normal genitalia and appendages,—in fact, all the essential organs of reproduction,—barrenness often results from enfeebled general health, gout, syphilis, excessive obesity, anemia, chronic alcoholism, and the like.

It is said the vagina, which is the natural receptacle for the male fluid, is bathed with an alkaline mucus in health, but from various diseased conditions this natural alkalinity is disturbed, and, as a result, the spermatozoon is killed. Such conditions are frequently seen in the various forms of vaginitis and particularly when of specific origin, which is most common in adult women. Hyperacidity is often due to general and constitutional causes, and must be corrected by means that will counteract them, such as the administration of alkalies and tonics, the employment of alkaline douches, the avoidance of stimulating foods and drinks, sexual abstinence; and, if possible, one or two daily bowel movements are to be encouraged by salines. One cannot say what degree of acidity is necessary to be lethal to the active ciliary movements of the spermatozoa, as some women become pregnant when the uterine and vaginal secretions are decidedly acid, as shown by litmus paper tests.

Leaving out the question of malformations and imperfect development of the vagina, various injuries are capable of producing sterility, such as extreme lacerations, which shorten and straighten the canal, elephantiasis, labial tumors, thick imperforate hymen and vaginismus.

Vaginismus is a quite common cause of infecundity; first of all, because it offers a great barrier to the proper performance of the sexual act; and, second, because the fissure, or erosion, or the painful caruncle which is so often the cause of it, keeps up the constantly irritating discharges. Within the past six months, I have treated a most obstinate case of vaginismus, first by forcible dilatation under chloroform, and then the removal of the hymen and its irritable carunculæ. Intercourse was still impossible, when I discovered a painful vaginal fissure which made the approaches of the husband intolerable. This I painted with a thirty-grain solution of silver nitrate. For another month graduated bougies were given the woman, and these were to be introduced daily into the vagina and left in position for a half an hour, until the spasm and pain were relieved. A perfect cure has resulted, and I presume pregnancy will soon follow. I have had a number of similar experiences during the past ten years.

The uterus may be defective in size, or absent, and as a result—sterility. Uterine stenosis is one of the most common causes of sterility,—first of all, on account of the obstruction it offers to the ready entrance of the spermatozoa; and, second, because of the mucus plug which usually fills up the canal, the result of associated endocervicitis and endometritis, due to the poor and deficient drainage.

In this class of cases, I am not content with the usual practice of treatment—namely, dilatation and curettage and packing with iodoform gauze. This I have found inadequate, and have used instead of the gauze a stem pessary, which is worn for weeks. These uteri are, as a rule, small and poorly developed; are often anteflexed, with the cervix long and pointed and, even when these uteri are thoroughly dilated, the canal very soon recontracts and closes to its original pinhole dimensions. I have observed a number of successful pregnancies to follow after this line of treatment; and I am satisfied that the stem not only provides a permanent opening, but gives tone and increases the growth and development of the uterus.

Let me detail the history of one case, which was operated upon previously. Mrs. V., aged 31 years, married six years, came to me the picture of health, but without children. Dr. Frederick curetted her two years before I saw her. Under chloroform I dilated the cervix, and curetted and painted the cavity of the uterus with one part of carbolic acid and two parts of tincture of iodine, and instructed her to wear a stem pessary for seven weeks, which was inserted at the time of the operation. It was then removed, and three months afterward she became pregnant. I delivered her with forceps, in due time, of a fine, healthy girl. Since then I have attended her for a boy, and have attended her in two provoked miscarriages.

Much harm can result from the careless and indiscriminate use of stem pessaries; but in a woman with healthy tubes and ovaries, and a movable uterus, no injury will follow their employment when provided with the slot on the side to favor drainage. I always instruct the woman to send for me if she complains of any pain and tenderness; and if she does not live in the city I instruct her how to remove the instrument at the first suggestion of discomfort.

Uterine deviations, with their associated catarrhs, are frequent causes of sterility or miscarriage, and particularly when the cervix is torn and turned out as a result of a previous labor. Curettage and drainage, and such plastic operations as may be necessary, and the Alexander operation for posterior displacements, when indicated, have brought me the most pleasant and most satisfactory results. I have now eleven patients upon whom I have performed the Alexander operation, who have conceived and were safely delivered, and in whom the uterus has remained in perfect position after confinement. Three of these never had children or a miscarriage previously, and two of them had had no children, one in five years, and the other in eleven years.

I am satisfied that many cases of endometritis have associated with them slight tubal disease, perhaps not sufficient to close the tube but, at all events, enough to lessen its elasticity, who are cured of their endometritis and tubal complication by a thorough curettage and iodoform pack,—cured symptomatically, and subsequently bear children.

Hyperinvolution is an occasional cause of sterility, and is seen in women who have borne a child. The uterus is very small and hard, and often only measures one and a half or two inches in size. Little can be done for this condition other than by electricity, in the form of intrauterine galvanisation, and the wearing of a stem pessary.

Tumors of the uterus are often a barrier to conception and subsequent pregnancy; and occasionally they can be removed even when gestation exists. In a paper written by Emmet, in the *American Gynecological and Surgical Journal,* June, 1900, he reports nine cases where myomectomy was performed on the pregnant uterus without inflicting any injury, and without causing premature expulsion of the ovum. Hysterectomy is not always necessary in cases of fibroid tumors of the uterus, for in many cases large tumors can be shelled out of their peritoneal covering; or, when attached by a pedicle, it can be cut and the uterus be left to functionate as though nothing had happened. Polyps and tumors presenting low down in the cervix likewise can be often easily removed.

Inflammations of the tubes and ovaries are very frequent causes of sterility. The tubes may be normal in size and appearance, and yet closed at their proximal end; or they may be enlarged and thickened by inflammatory exudate, or filled with pus, water or blood. They may be flexed, or so held out of position by adhesions as to make it impossible for their fimbriated ends to approximate to the rupturing Graffian follicle, and thus they do not receive the ovule; or their ciliary epithelial lining may be lost, so that the progress of the ovule is impeded, and then there may be developed an extrauterine pregnancy, if the ovum becomes segmented in the oviduct; or the ovaries may be so bound down and imbedded in adhesions that it is impossible for the Graffian follicle to rupture, or at all events to rupture at an exposed and accessible spot; or the ovarian tissue may be so disorganised as to be incapable of functionating, either from inflammation or the development of a neoplasm, solid or cystic.

Many conservative operations have been performed upon both tubes and ovaries, and pregnancy has followed. The tubes have been opened, or have been resected, and their mucous linings have been turned out. The ovaries have been resected, and even transplanted into the broad ligaments, and pregnancy has followed. It is surprising to see what nature has done with very small pieces of ovary and tubes, when they have been healthy and capable of functionating. Dr. Polk, of New York, reported a case where he removed an ovary from one side and the tube of the opposite side, and yet pregnancy took place, showing that the tube of the one side passed over to the ovary of the opposite side and picked up the ovule from its ruptured Graffian follicle. If this explanation is doubted, then it must be assumed that a small piece of ovary was left in the bite of the ligature, and yet so small that Dr. Polk overlooked it.

In one of my cases, we removed from a young unmarried woman the right tube and ovary for a large hydrosalpinx and ovarian cyst, and left the other tube and ovary, although it was pulled out of a nearly solid mass of inflammatory exudate. It appeared, however, reasonably healthy, although much bruised and swollen, so that the risk of leaving it seemed justifiable. Since the operation, she has married. and is now the mother of one boy and three beautiful girls.

Another interesting case was that of a young Italian woman, from whom Dr. Ingraham had removed the right tube and ovary. The left was healthy. I saw her two years after the operation, and found her suffering with an acutely retroverted movable uterus. I performed an Alexander operation upon her at the German Deaconess's Hospital, and since then Dr. Stein has attended her in two confinements, and she is now pregnant a third time. All this has occurred in less than four years, showing that one ovary and tube and a healthy uterus, are capable, under normal conditions, of multiplying and replenishing the earth so satisfactorily that race suicide, at all events among our foreign population, need not anxiously concern our statisticians and bureaus of vital statistics.

In view of such possibilities, and after reading the reports of conservative operations upon the appendages, by many different operators, it behooves us to leave as much of these organs as would be consistent, without inflicting too much possible risk and future suffering. We are not only to retain the important function of menstruation, but to encourage the possibility, if not probability, of pregnancy.

493 Delaware Avenue.

